# Prenatal arsenic exposure is associated with increased plasma IGFBP3 concentrations in 9-year-old children partly via changes in DNA methylation

**DOI:** 10.1007/s00204-018-2239-3

**Published:** 2018-06-08

**Authors:** Anda R. Gliga, Karin Engström, Maria Kippler, Helena Skröder, Sultan Ahmed, Marie Vahter, Rubhana Raqib, Karin Broberg

**Affiliations:** 1 Unit of Metals and Health, Institute of Environmental Medicine, Karolinska Institutet, 171 77 Stockholm, Sweden; 2 Division of Occupational and Environmental Medicine, Lund University, Lund, Sweden; 3 Division of Infectious Diseases, icddr,b, Dhaka, Bangladesh

**Keywords:** IGF1, IGFBP3, Epigenetic, Growth, Mediation, Early life

## Abstract

Exposure to inorganic arsenic (As), a carcinogen and epigenetic toxicant, has been associated with lower circulating levels of insulin-like growth factor 1 (IGF1) and impaired growth in children of pre-school age. The aim of this study was to assess the potential impact of exposure to As on IGF1 and insulin-like growth factor-binding protein 3 (IGFBP3) as well as DNA methylation changes in 9-year-old children. To this end, we studied 9-year-old children from a longitudinal mother–child cohort in rural Bangladesh (*n* = 551). Prenatal and concurrent exposure to As was assessed via concentrations in maternal urine at gestational week 8 and in child urine at 9 years, measured by HPLC-HG-ICPMS. Plasma IGF1 and IGFBP3 concentrations were quantified with immunoassays. DNA methylation was measured in blood mononuclear cells at 9 years in a sub-sample (*n* = 113) using the Infinium HumanMethylation450K BeadChip. In multivariable-adjusted linear regression models, prenatal As (natural log-transformed), but not children’s concurrent urinary As, was positively associated with IGFBP3 concentrations (*β* = 76, 95% CI 19, 133). As concentrations were not associated with IGF1. DNA methylation analysis revealed CpGs associated with both prenatal As and IGFBP3. Mediation analysis suggested that methylation of 12 CpG sites for all children was mediator of effect for the association between prenatal As and IGFBP3. We also found differentially methylated regions, generally hypermethylated, that were associated with both prenatal As and IGFBP3. In all, our study revealed that prenatal exposure to As was positively associated with IGFBP3 concentrations in children at 9 years, independent of IGF1, and this association may, at least in part, be epigenetically mediated.

**Electronic supplementary material:**

The online version of this article (10.1007/s00204-018-2239-3) contains supplementary material, which is available to authorized users.

## Introduction

Inorganic arsenic (As) is a well-established carcinogen (IARC 2012 100C) and a potent toxicant with effects on, e.g. the immune and endocrine systems (Ferrario et al. 2016; Raqib et al. 2017; Sun et al. 2016). Prenatal exposure to As has been adversely associated with changes in foetal size parameters (Kippler et al. 2012; Rahman et al. 2009; Yang et al. 2003) and exposure during early childhood has been associated with impaired growth (Gardner et al. 2013; Saha et al. 2012). Possibly related to this, prenatal exposure to As has been associated with lower concentrations of the plasma insulin-like growth factor 1 (IGF1), a marker of foetal and child growth, at birth (Ahmed et al. 2013). At 4.5 years of age, the children’s concurrent exposure to As, but not the prenatal exposure, was inversely associated with plasma IGF1 (Ahmed et al. 2013). Thus, the association between As and IGF1 during early life is unclear.

IGF1 is important for postnatal skeletal development, by mediating the anabolic and mitogenic activity of the pituitary growth hormone (Laron 2001), whereas during embryonic development IGF1 is regulating growth in a growth hormone-independent way (Cannata et al. 2010). In blood, 99% of IGF1 circulates in the form of heterotrimeric complexes with a binding protein and an acid-labile subunit that together mediate the tissue bioavailability of IGF1 (Jogie-Brahim et al. 2009). The most abundant binding protein in plasma is insulin-like growth factor-binding protein 3 (IGFBP3), which carries more than 75% of the circulating IGF1 (Jogie-Brahim et al. 2009). IGFBP3 also displays intrinsic activity, independent of IGF1, and has been reported to be involved in tumor development and progression by regulating cell growth and apoptosis (Baxter 2014). It is unknown if As can influence circulating concentrations of IGFBP3.

Expression of both IGF1 and IGFBP3 seems to be epigenetically regulated by DNA methylation. For IGF1, methylation of one of its promoters was found to be an epigenetic quantitative trait locus that modulates the amount of circulating IGF1 and partly explains the variation in human stature (Ouni et al. 2015). As regards IGFBP3, alterations of DNA methylation in the promoter have been associated with degree of expression and with survival rates from various cancer forms in humans (Perks and Holly 2015; Tomii et al. 2007).

Recent human studies have indicated that As exposure early in life may cause alterations of DNA methylation (reviewed in Argos 2015; Martin and Fry 2018). For example, we have reported associations between prenatal exposure to As and differentially methylated CpG sites in umbilical cord blood, especially of boys, with stronger effect for exposure in early versus late pregnancy (Broberg et al. 2014). In addition, prenatal exposure to As was found to be associated with changes in both DNA methylation and gene expression of 16 genes in cord blood leukocytes of Mexican infants (Rojas et al. 2015).

The objective of the present study was to evaluate the association between exposure to As (both prenatal and concurrent) and IGF1 as well as IGFBP3 concentrations in Bangladeshi children at 9 years of age. This was a follow-up of the children previously showing associations between As and IGF1 (Ahmed et al. 2013). We hypothesized that any effects of As on IGF1 and IGFBP3 are mediated through epigenetic regulation.

## Methods

### Study area and design

This study is part of our ongoing research in rural Bangladesh regarding the effects of arsenic and other metal contaminants in food and drinking water on pregnancy outcomes, child health and development (Ahmed et al. 2013; Gardner et al. 2013; Kippler et al. 2012; Vahter et al. 2006). The mother–child study sample in the present study was nested in a large randomized food and micronutrient supplementation trial conducted during pregnancy [Maternal and Infant Nutrition Interventions in Matlab (MINIMat)], in Matlab (a rural area, about 50 km southeast of Dhaka, Bangladesh) (Persson et al. 2012). In total, 4436 pregnant women were found to be eligible in the MINIMat trial (viable foetus, gestational age < 14 weeks, no severe illness, and consent for participation) and thereafter enrolled from November 2001 through October 2003. A sub-cohort (*n* = 1303) of the children born between June 2003 and June 2004 was selected to evaluate As-related immunotoxicity (Hawlader et al. 2013). Out of these children, 551 were followed up at 9 years of age and had donated urine as well as blood for the evaluation of exposure to As and plasma biomarkers. For a subset of 113 children with high quality DNA from peripheral blood mononuclear cells (PBMC), analysis of DNA methylation was performed by EWAS (epigenome-wide association study).

The study was approved by the research and ethics committees at icddr,b (Dhaka, Bangladesh) and the Ethical Review Board in Stockholm, Sweden. The study was conducted in accordance with the Helsinki Declaration. Informed consent was obtained from all participants, who were free to refrain from any part of the study at any time.

### Arsenic measurement

Arsenic exposure was evaluated based on the sum concentration of inorganic As species (arsenite and arsenate) and its methylated metabolites [methylarsonic acid (MMA) and dimethylarsinic acid (DMA)] in spot urine samples, measured by high-performance liquid chromatography coupled with hydride generation and inductively coupled plasma mass spectrometry (HPLC-HG-ICPMS) as previously described (Gardner et al. 2011; Skröder Löveborn et al. 2016). Maternal urine samples for prenatal As exposure were collected in early pregnancy between gestational weeks (GW) 5 and 14 (median GW 8) and child urine samples for concurrent exposure were collected at approximately 9 years of age. For the analyses of children’s urine, the limit of detection (LOD) was 0.2 µg/L for inorganic As(III), MMA and DMA, and 0.5 µg/L for inorganic As(V) (Skröder Löveborn et al. 2016). The LOD was 0.1 µg/L for As(III) and MMA and 0.2 µg/L for DMA and As(V) for the analysis of As during pregnancy (Gardner et al. 2011). All urine concentrations were adjusted for the average specific gravity (i.e. 1.012), to compensate for variation in urine dilution (Nermell et al. 2008).

### Measurement of IGF1 and IGFBP3

Venous blood samples were collected in Li-heparin-coated sterile vials (Becton Dickinson, Stockholm, Sweden) and plasma was separated by centrifugation, aliquoted and stored at − 80 °C. Plasma IGF1 was measured using the Human IGF1 ELISA kit (Quantakine ELISA, R&D Systems, Inc., Minneapolis, MN, USA) according to the manufacturer’s instructions. Absorbance was measured at 450 nm (reference 650 nm, wavelength correction set at 540) using a microplate reader and IGF1 concentrations were calculated based on standard curves. The LOD was 0.056 ng/mL, and the coefficient of variation was 5.6%. The plasma concentration of IGFBP3 was measured using Human IGFBP3 solid phase sandwich ELISA (Quantakine ELISA) according to the manufacturer’s instructions. The LOD was 0.14 ng/mL; the coefficient of variance of the assay was 6.2%.

### Measurement of DNA methylation

After plasma separation, the concentrated leukocyte band was collected and layered on top of Ficoll-Paque (Pharmacia-Upjohn/McNeill Laboratories, New Jersey, USA) and after density gradient centrifugation PBMC were separated. DNA was isolated from PBMC using QIAamp DNA Blood Midi kit (Qiagen, Hilden, Germany). The DNA quality was evaluated with a NanoDrop spectrophotometer (NanoDrop Products, Wilmington, DE, USA) and Bioanalyzer 2100 (Agilent, Santa Clara, CA, USA) and showed good quality (260/280 nm > 1.80). 250 ng of DNA was bisulfite-treated with the EZ DNA Methylation kit (Zymo Research, Irvine, CA, USA). For each sample, the bisulfite converted DNA was eluted in 30 µL, which was further evaporated to a volume of < 4 µL, and used for methylation analysis using the Infinium HumanMethylation 450k BeadChip (Illumina, San Diego, CA, USA). The methylation analysis was performed at SciLifeLab, Uppsala, Sweden. All beadchips were from the same batch. Image processing, background correction, quality control, filtering, and normalization (by the SWAN procedure) were performed in the R package *minfi* (Aryee et al. 2014; Maksimovic et al. 2012). The 450k array included a total of 485,512 sites before filtering. All samples performed well and had at least 98% of the CpGs with detection *p* value below 0.01. We removed CpGs for which more than 20% of the samples had a detection *p* value above 0.01 (*n* = 322). Furthermore, the following probes were removed: rs probes and CpH probes (*n* = 3091), probes with in silico nonspecific binding (*n* = 29,118), probes on the X and Y chromosomes (*n* = 10,329), and probes with common single nucleotide polymorphisms (according to the function dropLociWithSnps in minfi; *n* = 15,424). In total, 426,936 probes were left for further analysis. For the analyses stratified by sex, X and Y chromosomes were included which amounted to a total of 437,179 probes.

### Covariates

Description of the characteristics for this cohort was previously reported (Persson et al. 2012; Raqib et al. 2017). Maternal anthropometry and parity, as well as socioeconomic status (SES) of the families were collected in early pregnancy at the enrolment in the MINIMat trial (Persson et al. 2012). SES was estimated via an asset index, generated through principal component analysis of household assets (Gwatkin et al. 2007). The SES of the family was updated during the follow-up of the children at 9 years of age. Body weight was measured to the nearest 0.1 kg by a digital scale (TANITA HD-318; Tanita Corporation, Japan) and a standard weight of 20 kg was used to calibrate the digital scale regularly. Height was measured with a free-standing stadiometer [Leicester Height Measure (nearest to 0.1 cm; Seca 214, UK)]. The measured height and weight were converted to height-for-age and weight-for-age z-scores (i.e. HAZ and WAZ), using the WHO growth reference for school-aged children and adolescents (de Onis et al. 2007). Season of birth and season of blood collection were categorized as pre-monsoon (January–May), monsoon (June–September), and post-monsoon (October–December). Plasma C-reactive protein (CRP), a marker of acute inflammation, was evaluated in the children at 9 years of age, as previously described (Raqib et al. 2017) and was regarded as potential covariate, as inflammation has been shown to interfere with the IGF1 axis (Wong et al. 2016). In addition, for the DNA methylation analysis, predicted cell counts were regarded as covariates (as described below in the Statistical analyses, “Bioinformatic analyses of the DNA methylation data”).

### Statistical analyses

#### Associations between exposure to As and plasma biomarkers

Differences in maternal and child characteristics between the EWAS sub-sample (*n* = 113) and the whole study sample (*n* = 551), as well as sex differences, were evaluated by *t* test and Mann–Whitney–Wilcoxon test (normality was evaluated by Shapiro–Wilk test).

We used linear regression analyses to investigate associations of exposure to As (maternal urinary As in early pregnancy and concurrent urinary As at 9 years) with plasma concentrations of IGF1 and IGFBP3, as well as with the molar ratio of IGF1 and IGFBP3, which reflects the bioavailability of IGF1. The analyses were also stratified by sex, since there were significant differences between boys and girls in terms of plasma IGF1 and IGFBP3 concentrations in this study sample, in line with a previous study on European children (Geary et al. 2003).

Model assumptions were evaluated by whether the residuals had constant variance, were normally distributed, and had an approximate linear relationship with each continuous covariate. Exposure variables (As in urine at GW 8 and at 9 years) were natural log (ln)-transformed to meet model assumptions. Regression models were constructed with covariates that were associated with exposure and/or outcome based on Spearman rank correlation coefficients (for continuous variables) or Kruskal–Wallis (for categorical variables). All considered covariates were kept in the final model if they modified the effect estimate by more than 10% by backwards elimination. For IGF1, the covariates considered were sex, HAZ (at 9 years), SES (GW 8 and 9 years), total IgE (9 years), BMI (GW 8 and 9 years), maternal age, maternal education, parity, CRP, season of blood collection and IGFBP3. For IGFBP3, the covariates considered were sex, HAZ (9 years), IGF1, CRP and season of blood collection. HAZ was included in the model as a covariate (marker of nutritional status) but it was also considered as an outcome (marker of growth), evaluated by Spearman correlation.

To evaluate independent effects of IGF1 and IGFBP3, each protein was included as covariate in the multivariable adjusted models of the other respective protein. For comparison, we also constructed models where we excluded IGF1 and IGFBP3 as covariates. In addition, we investigated the associations between exposure to As and the molar ratio IGF1/IGFBP3 using this as the outcome.

Since maternal urinary As was correlated with As in urine at 9 years, we constructed a model including As in urine at 9 years to evaluate whether the association between maternal urinary As and IGFBP3 was biased by the As in urine at 9 years.

We also performed mediation regression analysis (Küpers et al. 2015) to evaluate whether DNA methylation could be a mediator of any association of prenatal As exposure and IGFBP3 concentrations in plasma. The mediation analysis was run on the EWAS subset of 113 individuals as follows: (1) first we identified CpG sites of interest in the *IGFBP3* gene as well as sites that were associated with both prenatal As and IGFBP3 concentrations (top 2000 CpG sites were evaluated). (2) Next, we performed linear regression analyses (crude and multivariable-adjusted) to evaluate the association between prenatal As exposure and IGFBP3 in plasma in this sample subset of 113 individuals. (3) Finally, the CpG sites from (1) for which the methylation was associated with both prenatal As exposure and IGFBP3 concentrations were included as covariates (one by one) in the multivariable-adjusted models from step (2). Methylation was considered a mediator if it resulted in > 10% attenuation of the model (measured as the effect estimate for As) (Anderson et al. 2017), comparing the multivariable-adjusted models with and without adjusting for methylation. A scheme of the mediation analysis is provided in the Figure S1.

All statistical analyses were performed using R (v.3.4.1). *p* values < 0.05 were considered significant, unless otherwise stated.

#### Bioinformatic analysis of the DNA methylation data

For the association between prenatal As and DNA methylation, we focused on As exposure in early gestation, as DNA methylation in cord blood was previously reported to have a stronger association with exposure to As in early (GW 8) as compared with late (GW 30) gestation (Broberg et al. 2014). In addition, we evaluated the association between DNA methylation and IGFBP3 concentrations in plasma. Analyses were performed including all individuals as well as after stratifying by sex.

The cell composition of mononuclear cells could impact the DNA methylation. In the 9 year-old children, we estimated the cell type proportions using the *minfi* estimateCellCounts function, which is based on the method developed by Houseman et al. (2012) and considers adult white blood cells as a reference (Houseman et al. 2012). This method was shown to be also valid for school-aged children (12 years) (Yousefi et al. 2015). DNA methylation was adjusted for technical variation, via ComBat adjustment (Johnson et al. 2007). Principal component analysis (PCA) was performed on DNA methylation values expressed as ComBat-adjusted *M* values, using the universally applicable singular value decomposition, to evaluate the influence of biological variables. The PCA showed that estimated cell counts influenced the DNA methylation, and the cell types most strongly associated with DNA methylation (monocytes and granulocytes) were included as adjustment variables in the analyses of DNA methylation in relation to As or IGFBP3.

Differently methylated positions (DMPs) in relation to prenatal As exposure (ln-transformed) and IGFBP3 concentrations at 9 years were evaluated by fitting a robust linear regression model to each CpG (*M* values) using the R package *limma* where empirical Bayes smoothing was applied to the standard errors (Ritchie et al. 2015). Sex of the child was included as an adjustment variable in the analyses comprising all individuals along with the estimated cell counts (monocytes and granulocytes). *p* values were adjusted for false discovery rate (FDR) to obtain *q* values and a *q* value of 0.05 or lower was considered statistically significant. There were no overlapping sites between the two EWAS analyses (prenatal As and IGFBP3) when we considered a *q* < 0.05 and that is likely related to the high stringency of the two analyses as they were both adjusted for multiple testing. For this reason, we evaluated the overlap between the top 2000 CpGs to identify CpGs that could act as mediators (mediation analysis is described in the previous section) and we found a total of 14 sites that were associated with both prenatal As and IGFBP3 (unadjusted *p* value < 0.005) in all children. In the sex-stratified analysis, we identified 11 CpG sites in boys and girls, each.

Differently methylated regions (DMRs) were evaluated using the *bumphunter* function in the R package *minfi* (Jaffe et al. 2012) on the ComBat-adjusted beta values, using the smoothing function, 1000 permutations and a maximum gap between the CpGs of 300 base pairs. After the analysis, we introduced a threshold of at least four CpG sites per DMR.

Venn diagrams were generated using a web-based tool developed by the Bioinformatics and Evolutionary Genomics Laboratory at VIB/UGent, Belgium (http://bioinformatics.psb.ugent.be/webtools/Venn/). Pathway and network analyses were performed using Ingenuity Pathway Analysis (content version 39480507, license obtained from Ingenuity Systems, Redwood City, CA, USA) on the genes denoted by the top 500 DMPs based on *p* value. The top 500 CpG sites that correlated with prenatal As exposure in all children, as well as after stratification by sex, were annotated to the corresponding genes that constituted input data for the pathway/network analysis. In addition, we performed overrepresentation enrichment analysis for the genes corresponding to the overlapping DMRs between prenatal As and IGFBP3 in boys, in terms of cytogenic bands using the Webgestalt platform (Wang et al. 2017). All bioinformatic analyses were performed using R (v.3.4.1), unless otherwise stated.

## Results

The characteristics of the studied children are summarized in Table 1. In the whole study sample (*n* = 551), boys had slightly higher BMI as compared to girls, while girls had higher values of plasma marker of inflammation (CRP), IGF1 and IGFBP3 (*p* for all < 0.05). For the subset of individuals used in the EWAS analysis, sex differences were retained for plasma IGF1. There were no significant differences in the characteristics between the whole study sample (*n* = 551) and the EWAS sub-sample (*n* = 113).

**Table 1:** Characteristics of the 9-year-old children included in the study

Variables	Study sample				EWAS sub-sample				*p* value^b^
	All children *n* = 551	Boys *n* = 275	Girls *n* = 276	*p* value^a^	All children *n* = 113	Boys *n* = 50	Girls *n* = 63	*p* value^a^	
Age (years)	8.90 ± 0.13	8.89 ± 0.11	8.90 ± 0.14	0.857	8.89 ± 0.14	8.87 ± 0.09	8.91 ± 0.16	0.492	0.361
HAZ	− 1.36 ± 0.88	− 1.30 ± 0.91	− 1.41 ± 0.85	0.133	− 1.43 ± 0.93	− 1.26 ± 1.01	− 1.56 ± 0.85	0.100	0.466
WAZ	− 1.74 ± 1.05	− 1.72 ± 1.07	− 1.75 ± 1.04	0.662	− 1.72 ± 1.09	− 1.74 ± 1.11	− 1.71 ± 1.07	0.861	0.979
BMI GW 8 (kg/m^2^)	20.1 (14.3, 35.3)	20.1 (15.4, 30.3)	19.9 (14.3, 35.3)	0.324	19.9 (15.4, 29.8)	21.1 (15.4, 29.2)	19.7 (17.1, 29.8)	0.036	0.546
BMI 9 years (kg/m^2^)	14.0 (6.3, 25.4)	14.1 (11.1, 22.8)	13.9 (6.3, 25.4)	0.036	14.1 (11.1, 23.6)	13.9 (11.1, 17.9)	14.2 (11.2, 23.6)	0.452	0.473
SES (GW 8)	0.88 (− 5.82, 3.75)	0.93 (− 5.55, 3.75)	0.81 (− 5.82, 3.50)	0.896	1.12 (− 5.82, 3.65)	1.20 (− 5.14, 3.65)	0.71 (− 5.82, 3.38)	0.297	0.567
SES (9 years)	− 0.17 (− 2.03, 2.92)	− 0.17 (− 1.96, 2.87)	− 0.17 (− 2.03, 2.92)	0.996	− 0.11 (− 1.96, 2.92)	− 0.02 (− 1.96, 2.72)	− 0.23 (− 1.80, 2.92)	0.284	0.580
Plasma CRP (mg/L)	0.36 (0.03, 22.64)	0.33 (0.03, 14.56)	0.40 (0.07, 22.64)	0.013	0.42 (0.08, 14.8)	0.40 (0.08, 11.41)	0.46 (0.10, 14.8)	0.343	0.185
Plasma IGF1 (ng/mL)	93 (30, 245)	83 (30, 174)	103 (34, 245)	< 0.001	95 (38, 197)	79 (39, 161)	102 (38, 197)	< 0.001	0.948
Plasma IGFBP3 (ng/mL)	2769 (611, 6485)	2633 (611, 5673)	2923 (1130, 6485)	< 0.001	2660 (1130, 4527)	2654 (1200, 4527)	2683 (1130, 4485)	0.333	0.619
As in urine (µg/L) GW 8^c^	77 (2, 2064)	72.5 (2, 1535)	95.0 (4, 2064)	0.198	68 (16, 679)	62 (17, 679)	75 (16, 551)	0.474	0.362
As in urine (µg/L) 9 years^c^	53 (9, 1268)	51 (9, 1268)	53 (12, 648)	0.099	48 (10, 481)	43 (10, 332)	57 (14, 481)	0.056	0.700

Data are presented as mean ± SD or median and range

*GW* gestational week, *HAZ* height-for-age *z*-scores, *SES* socioeconomic status, *CRP* C-reactive protein, *IGF1* insulin-like growth factor 1, *IGFBP3* insulin-like growth factor-binding protein 3

^a^*p* values testing the differences between boys and girls for the respective dataset using Student’s *t* test or Mann–Whitney–Wilcoxon test, as appropriate (normality evaluated by Shapiro–Wilk test)

^b^*p* values testing the differences between the two datasets (whole cohort and EWAS) using Student’s *t* test or Mann–Whitney–Wilcoxon test, as appropriate (normality evaluated by Shapiro–Wilk test)

^c^Adjusted for average specific gravity (1.012)

### Urinary arsenic in early pregnancy is positively associated with IGFBP3, but not with IGF1, concentrations in 9-year-old children

HAZ and WAZ were moderately correlated with plasma concentrations of IGF1 (*r*_S_ = 0.36, *p* < 0.001; *r*_S_ = 0.41, *p* < 0.001) and IGFBP3 (*r*_S_ = 0.22, *p* < 0.001; *r*_S_ = 0.20, *p* < 0.001), but they were not correlated with either prenatal (*r*_S_ = − 0.006, *r*_S_ = − 0.001) or concurrent As exposure (*r*_S_ = − 0.014, *r*_S_ = − 0.003). IGF1 and IGFBP3 were positively inter-correlated (*r*_S_ = 0.47, *p* < 0.001).

**Fig. 1:**
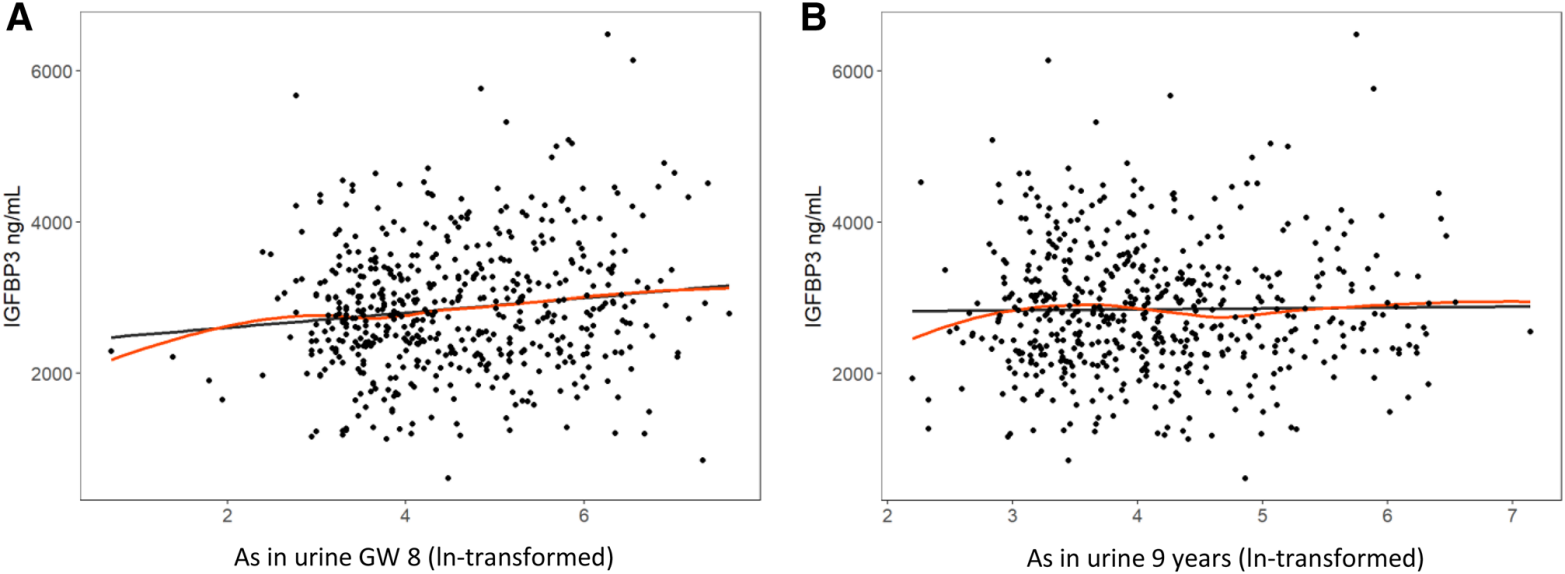
Prenatal arsenic (As) is associated with plasma concentrations of IGFBP3 in 9-year-old children. Prenatal (**a**) but not concurrent (**b**) exposure to As is associated with IGFBP3 plasma concentrations in 9-year-olds. Prenatal exposure corresponds to natural log-transformed maternal As in urine in gestational week 8 [ln(As in urine GW 8)], and concurrent exposure to natural log-transformed As in urine at 9 years [ln(As in urine 9 years)]. Black line indicates the linear regression line (crude model) and red line indicates the Loess line

Using linear regression models, we evaluated associations between prenatal and concurrent As exposure (both natural log-transformed) and the plasma concentrations of IGF1, IGFBP3 and their respective molar ratio in the 9-year-olds (Table 2; Fig. 1). IGF1 modified the effect estimate for As in the model analysing the association between exposure to As and IGFBP3, and vice versa. In the crude model, there was a non-significantly positive association between maternal urinary arsenic in early pregnancy (hereinafter called prenatal As) and IGF1 for all children (*β* = 2.2, 95% CI − 0.3, 4.7) and for girls (*β* = 3.1, 95% CI − 0.6, 6.8), but no associations were found in the multivariable adjusted model. The associations in the crude models were largely influenced by IGFBP3; the addition of IGFBP3 as single covariate to the crude model modified the effect estimate from 2.2 to 0.5 in all children and from 3.1 to 1.9 in girls. No association was found between concurrent urinary As and IGF1 (Table 2).

**Table 2:** Multivariable adjusted linear regression analysis of ln-transformed urinary As concentrations with plasma concentrations of IGF1 and IGFBP3 at 9 years

Outcome	Exposure	All children *β* (95% CI); *p* value	Boys *β* (95% CI); *p* value	Girls *β* (95% CI); *p* value
IGF1 (ng/mL)	As in urine at GW 8 (ln-transformed)			
	Crude model (*n* = 535/266/269)	2.2 (− 0.3, 4.7); 0.089	− 0.1 (− 3.1, 3.0); 0.962	3.1 (− 0.6, 6.8); 0.098
	Adjusted model^a^ (*n* = 513/255/258)	1.2 (− 1.0, 3.4); 0.285	− 0.1 (− 2.9, 2.6); 0.928	2.4 (− 1.0, 5.8); 0.169
	Adjusted model^b^ (*n* = 513/255/258)	0.1 (− 2.0, 2.1); 0.946	− 1.4 (− 3.9, 1.0); 0.256	1.5 (− 1.9, 4.8); 0.388
	As in urine at 9 years (ln-transformed)			
	Crude model (*n* = 548/273/275)	1.0 (− 2.3, 4.2); 0.559	− 0.3 (− 4.1, 3.5); 0.868	0.7 (− 4.1, 5.5); 0.779
	Adjusted model^c^ (*n* = 535/267/268)	0.4 (− 2.3, 3.0); 0.795	0.5 (− 2.8, 3.9); 0.750	− 0.1 (− 4.3, 4.2); 0.980
IGFBP3 (ng/mL)	As in urine at GW 8 (ln-transformed)			
	Crude model (*n* = 535/266/269)	98 (36, 160); 0.002	86 (0, 171); 0.050	95 (6, 183); 0.036
	Adjusted model^d^ (*n* = 524/261/263)	91 (29, 153); 0.004	84 (− 3, 171); 0.057	97 (8, 187); 0.034
	Adjusted model^e^ (*n* = 524/261/263)	76 (19, 133); 0.009	85 (12, 159); 0.024	76 (− 10, 162); 0.082
	As in urine at 9 years (ln-transformed)			
	Crude model (*n* = 548/273/275)	12.3 (− 66, 91); 0.758	− 1.6 (− 107, 104); 0.976	5.1 (− 109, 119); 0.930
	Adjusted model^d^ (*n* = 537/268/269)	0.9 (− 78, 79); 0.983	− 1.6 (− 109, 106); 0.976	5.6 (− 110, 121); 0.924
	Adjusted model^e^ (*n* = 537/268/269)	− 1.1 (− 72, 70); 0.975	− 0.4 (− 90, 91); 0.993	1.6 (− 107, 111); 0.976
IGF1/IGFBP3	As in urine at GW 8 (ln-transformed)			
	Crude model (*n* = 535/266/269)	− 0.001 (− 0.005, 0.003); 0.585	− 0.004 (− 0.008, 0.000); 0.078	0.001 (− 0.005, 0.006); 0.793
	Adjusted model^f^ (*n* = 513/255/258)	− 0.002 (− 0.006, 0.001); 0.250	− 0.004 (− 0.008, 0.000); 0.065	0.000 (− 0.006, 0.006); 0.935
	As in urine at 9 years (ln-transformed)			
	Crude model (*n* = 548/273/275)	0.001 (− 0.004, 0.005); 0.729	− 0.001 (− 0.007, 0.004); 0.650	0.002 (− 0.005, 0.009); 0.653
	Adjusted model^g^ (*n* = 535/267/268)	0.001 (− 0.004, 0.005); 0.798	0.000 (− 0.005, 0.005); 0.970	0.001 (− 0.006, 0.008); 0.789

^a^Adjusted for child sex (except when stratified for sex), HAZ (9 years), BMI (GW 8), plasma CRP (9 years)

^b^Adjusted for child sex (except when stratified for sex), HAZ (9 years), BMI (GW 8), plasma CRP (9 years), plasma IGFBP3

^c^Adjusted for child sex (except when stratified for sex), HAZ (9 years), BMI (9 years), plasma CRP (9 years), SES (9 years)

^d^Adjusted for child sex (except when stratified for sex), plasma CRP (9 years)

^e^Adjusted for child sex (except when stratified for sex), plasma CRP (9 years), plasma IGF1

^f^Adjusted for child sex (except when stratified for sex), HAZ (9 years), plasma CRP (9 years), BMI (GW 8)

^g^Adjusted for child sex (except when stratified for sex), HAZ (9 years), plasma CRP (9 years), SES (9 years), BMI (9 years)

Prenatal As was positively associated with IGFBP3 concentrations in plasma (Table 2; Fig. 1). This association was present both in the crude (*R*^2^ = 0.01, *β* = 98, 95% CI 36, 160) and in the multivariable adjusted models (with IGF1 as a covariate *R*^2^ = 0.20, *β* = 76, 95% CI 19, 133 and without *R*^2^ = 0.04, *β* = 91, 95% CI 29, 153). If we further adjusted the latter model for child urinary As at 9 years, the effect estimate increased by approximately 7% (*β* = 97). When we stratified by sex, the association was maintained in the crude model for both boys (*β* = 86, 95% CI 0.1, 171) and girls (*β* = 95, 95% CI 6, 183). In the multivariable-adjusted models, the effect estimates for the association between IGFBP3 and prenatal As were largely similar as compared with the respective crude models, and between boys and girls (Table 2). For interpretation purposes, an increase in the maternal As concentrations in urine by 100 µg/L was associated with an increase in the children’s IGFBP3 by 33 ng/mL, corresponding to 4% of the standard deviation (SD for IGFBP3 = 842). There were no associations between concurrent As exposure and IGFBP3 concentrations (Table 2; Fig. 1b).

In the multivariable-adjusted regression models (As versus IGF1 or IGFBP3), the number of individuals decreased due to missing data for some of the covariates as compared with the crude models (Table 2). In the sensitivity analyses, we re-run the crude models with only the individuals used in the multivariable adjusted analyses and there was no change in the significance levels.

Finally, we investigated the associations between arsenic exposure and the molar ratio of IGF1/IGFBP3, which is reported to measure the bioavailability of IGF1 (Jogie-Brahim et al. 2009). A negative association was found in boys, but it was not significant (Table 2).

### Maternal urinary arsenic during pregnancy is associated with methylation of the *IGFBP3* promoter

**Fig. 2:**
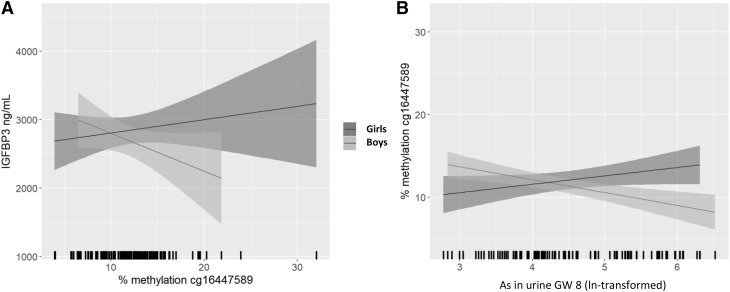
Methylation of cg16447589 is associated with IGFBP3 concentrations in plasma and prenatal arsenic exposure. **a** Methylation of cg16447589 is negatively associated with IGFBP3 concentrations in plasma in boys and positively associated in girls. **b** Prenatal exposure to As, expressed as natural log-transformed maternal arsenic in urine in gestational week 8 [ln(As in urine GW 8)], is negatively associated with methylation of cg16447589 in boys and positively associated in girls. Lines indicate the regression line, and the shadows represent the 95% confidence interval

We evaluated whether the associations between prenatal As and IGFBP3 in plasma were epigenetically mediated by first looking into the DNA methylation of sites within the *IGFBP3* gene. There are 39 CpG sites on *IGFBP3* that passed the quality control for the EWAS analysis, out of which 28 CpG sites are within the promoter region and 11 sites are intragenic (Table S1). Using linear regression models, we evaluated the association between the methylation (%, ComBat-adjusted) of the respective sites and the IGFBP3 concentrations with a *p* value of 0.1 as threshold for significance. In total, 13 CpG sites were associated with IGFBP3 and out of these CpG sites, one was associated with both IGFBP3 and prenatal As, but differently depending on the sex (Table S1, Table 3): methylation of cg16447589 was associated with lower IGFBP3 in boys (*β* = − 61, 95% CI − 128, 5), but not in girls (*β* = 22, 95% CI − 24, 68; Fig. 2a). The same cg16447589 was inversely associated with prenatal As in boys (*β* = − 1.67, 95% CI − 2.52, − 0.81), but positively in girls (*β* = 1.20, 95% CI 0.01, 2.38; Table 3; Fig. 2b). Adjusting for methylation of cg16447589 (mediation analysis) in the multivariable-adjusted model for prenatal As versus IGFBP3 (boys) in the EWAS sub-sample, decreased the estimate from *β* = 152 (95% CI − 54, 359, *p* = 0.144) to *β* = 121 (95% CI − 117, 359, *p* = 0.411), which amounts to 20% mediation (Table S5). This suggests that methylation of cg16447589 could partly act as a mediator for the association between prenatal As and circulating IGFBP3 at 9 years in boys. Analogous analysis in girls was not meaningful due to the very low effect estimate in this sub-sample (*β* = 11) for prenatal As versus IGFBP3.

**Table 3:** Linear regression analysis of methylation of CpG sites within the *IGFBP3* gene in mononuclear cells at 9 years with maternal urinary As (GW 8, ln-transformed)

CpG site in *IGFBP3*	Location in *IGFBP3*	All children (*n* = 112) *β* (95% CI); *p* value	Boys (*n* = 49) *β* (95% CI); *p* value	Girls (*n* = 63) *β* (95% CI); *p* value
cg08541297	Promoter	− 0.16 (− 0.58, 0.27); 0.476	− 0.60 (− 1.18, − 0.02); 0.042	0.16 (− 0.49, 0.80); 0.630
cg04690927	Promoter	0.02 (− 0.26, 0.31); 0.880	0.17 (− 0.28, 0.64); 0.441	− 0.15 (− 0.55, 0.24); 0.445
cg13977557	Promoter	0.14 (− 0.02, 0.30); 0.087	0.19 (− 0.04, 0.42); 0.102	0.09 (− 0.14, 0.33); 0.427
cg16447589	Promoter	− 0.14 (− 0.94, 0.65); 0.722	− 1.67 (− 2.52, − 0.81); 0.000	1.20 (0.01, 2.38); 0.047
cg06789764	Promoter	0.13 (− 0.26, 0.53); 0.496	− 0.28 (− 0.87, 0.30); 0.341	0.45 (− 0.11, 1.02); 0.114
cg26434048	Promoter	− 0.21 (− 0.98, 0.55); 0.581	− 0.04 (− 1.04, 0.94); 0.921	− 0.45 (− 1.65, 0.74); 0.450
cg16875425	Intragenic	0.45 (− 0. 37, 1.27); 0.278	0.95 (− 0.28, 2.18); 0.128	0.19 (− 0.95, 1.32); 0.740
cg03776080	Intragenic	− 0.23 (− 0.70, 0.24); 0.342	− 0.17 (− 0.72, 0.38); 0.534	− 0.28 (− 1.05, 0.49); 0.473
cg23193639	Intragenic	0.20 (− 0.27, 0.68); 0.406	− 0.31 (− 1.04, 0.41); 0.386	0.58 (− 0.08, 1.24); 0.084
cg22083798	Intragenic	− 0.16 (− 0.98, 0.66); 0.700	− 0.097 (− 1.35, 1.15); 0.877	− 0.36 (− 1.15, 0.75); 0.521
cg02120774	Intragenic	− 0.003 (− 0.33, 0.33); 0.982	− 0.28 (− 0.76, 0.20); 0.251	0.14 (− 0.34, 0.62); 0.559
cg11753867	Intragenic	0.14 (− 0.07, 0.45); 0.193	0.18 (− 0.12, 0.49); 0.237	0.12 (− 0.18, 0.43); 0.420
cg20850023	Intragenic	− 0.06 (− 0.52, − 0.39); 0.780	− 0.34 (− 1.01, − 0.34); 0.320	− 0.13 (− 0.49, − 0.76); 0.674

Methylation data were derived from the ComBat-adjusted % methylation values (excluding sites from X and Y chromosomes for analysis on all children, and including sites from X and Y chromosomes for sex-stratified analysis). The CpG sites were selected from the sites present on *IGFBP3* and were associated with prenatal exposure to As. The linear models were adjusted for child sex (except when stratified for sex), estimated granulocytes and monocytes

### Prenatal arsenic and IGFBP3 are associated with differentially methylated positions (DMPs)

**Fig. 3:**
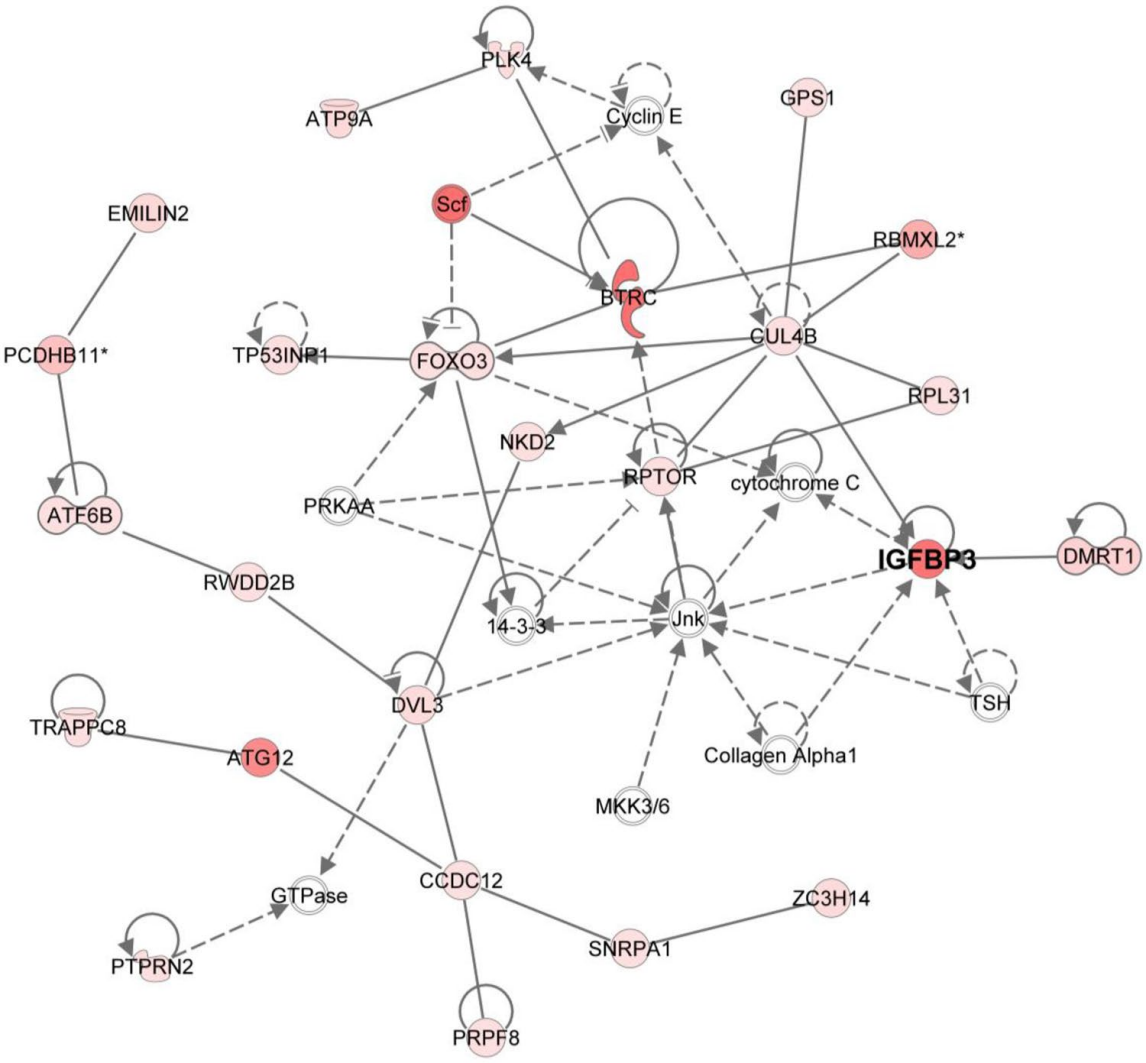
Top network of the genes defined by the differentially methylated CpG sites associated with the prenatal exposure to arsenic in boys (IGFBP3 was annotated for the CpG cg16447589). The main diseases and functions associated with this network are related to cancer, organismal injuries and abnormalities, melanoma and cell cycle progression. Asterisk indicates genes for which more than one CpG had been annotated

To further understand whether the relation between prenatal As and IGFBP3 in plasma could be epigenetically mediated, we performed differential methylation analysis (*limma*), using the prenatal As as an independent variable. We identified 9 DMPs for all individuals, 57 for boys (one in boys was the aforementioned *IGFBP3* cg16447589, Table S3) and 15 for girls (Tables S2, S3). The main network defined by the top 500 CpGs in boys contained the cg16447589 (Fig. 3) and was associated with cancer, organismal injury and abnormalities, as well as with melanoma and cell cycle progression. However, it should be noted that IGF1 is not part of this network.

Next we identified top CpG sites that were associated with both prenatal As and IGFBP3 concentrations which could act as potential mediators (Table S5). For all children, we identified 14 overlapping sites out of which 12 were found as mediators, for boys we identified 11 overlapping sites out of which all were found to be mediators of effect (in total 12 sites with *IGFBP3* cg16447589; Table S5). Analogous analysis in girls was not meaningful due to the very low effect estimate (*β* = 11) for prenatal As versus IGFBP3 in this sub-sample.

### Prenatal arsenic and IGFBP3 are associated with differentially methylated regions (DMRs)

Analysis of DMRs associated with prenatal As and with IGFBP3 (Table 4) revealed 10 overlapping DMRs for all children, 26 overlapping DMRs for boys and 5 overlapping DMRs for girls. In general, these regions were mostly hypermethylated and located in regulatory regions, i.e. near histone acetylation marks and with binding motifs for transcription factors (as per UCSC Genome Browser). The top overlapping DMR for all children as well as for boys was hypermethylated, located on the *MIR886* gene in a regulatory region that is rich in transcription factor (e.g. JUN, FOS, EP300, RELA) binding sites and bears a histone acetylation mark (H3K27). A full list of DMRs for prenatal As is included in Table S6. Cytogenic band enrichment analysis for the genes corresponding to the overlapping DMRs in boys indicated significant enrichment (FDR-adjusted *p* value < 0.05) for chromosome X in Xp11.23, Xq28 and Xq24.

**Table 4:** Overlapping differentially methylated regions (DMRs) in peripheral blood mononuclear cells at 9 years in relation to maternal urinary As (GW 8, ln-transformed) and plasma IGFBP3 concentrations at 9 years

Overlap			Prenatal As					IGFBP3				
Chr	Gene symbol	Gene name	Start	End	*L*	*p* value	Dir.	Start	End	*L*	*p* value	Dir.
All												
5	*MIR886 (VTRNA2-1)*	Vault RNA 2-1	135,415,693	135,416,613	16	0.001	+	135,415,693	135,416,613	16	0.004	+
17	*SLFN12*	Schlafen family member 12	33,759,484	33,760,419	12	0.002	+	33,759,484	33,760,293	11	0.007	−
16	*ABAT*	4-aminobutyrate aminotransferase	8,806,531	8,807,018	10	0.010	−	8,806,531	8,806,966	8	0.037	−
5	NA	NA	1,867,978	1,868,639	5	0.026	+	1,867,978	1,868,738	7	0.005	+
11	NA	NA	67,383,377	67,383,862	7	0.040	+	67,383,377	67,384,040	8	0.005	+
1	*OR2L13*	Olfactory receptor family 2 subfamily L member 13	248,100,345	248,100,614	7	0.015	−	248,100,228	248,100,614	9	0.002	+
17	*LOC728392*	NA	5,402,972	5,403,516	5	0.026	−	5,403,053	5,403,516	4	0.015	+
19	*AURKC*	Aurora kinase C	57,742,217	57,742,444	8	0.028	+	57,742,112	57,742,444	9	0.024	−
11	*LDHC*	Lactate dehydrogenase C	18,433,564	18,433,887	5	0.031	−	18,433,500	18,433,745	6	0.001	+
1	*S100A13*	S100 calcium-binding protein A13	153,599,671	153,600,156	6	0.037	+	153,599,479	153,600,156	8	0.010	+
Boys												
5	*MIR886 (VTRNA2-1)*	Vault RNA 2-1	135,415,693	135,416,613	16	0.000	+	135,415,693	135,416,613	16	0.003	+
X	*NDUFB11* *RBM10*	NADH:Ubiquinone oxidoreductase subunit B11 RNA-binding motif protein 10	47,004,051	47,005,131	18	0.006	+	47,004,051	47,005,131	18	0.005	+
X	*IKBKG*	Inhibitor of nuclear factor kappa B kinase subunit gamma	153,775,262	153,776,009	16	0.010	+	153,775,262	153,776,009	16	0.007	+
17	*SLFN12*	Schlafen family member 12	33,759,484	33,760,419	12	0.013	+	33,759,484	33,760,293	11	0.013	−
X	*RNF113A*	Ring finger protein 113A	119,005,413	119,006,122	14	0.015	+	119,005,413	119,006,122	14	0.008	+
X	*ZNF674*	Zinc finger protein 674	46,404,528	46,405,125	14	0.015	+	46,404,528	46,405,125	14	0.010	+
X	*UPF3B*	Regulator of nonsense mediated MRNA decay	118,986,897	118,987,328	13	0.019	+	118,986,703	118,987,328	14	0.009	+
X	*LOC100272228*	NA	149,106,454	149,107,029	13	0.021	+	149,106,022	149,107,029	15	0.010	+
X	*TAZ* *DNASE1L1*	Tafazzin Deoxyribonuclease 1 like 1	153,639,778	153,640,967	12	0.026	+	153,639,778	153,640,967	12	0.013	+
X	*HMGB3*	High mobility group box 3	150,151,580	150,151,823	11	0.029	+	150,151,572	150,151,823	12	0.016	+
X	*RPL36A*	Ribosomal protein L36a	100,645,695	100,646,162	11	0.037	+	100,645,695	100,646,162	11	0.020	+
X	*FAM104B*	Family with sequence similarity 104 member B	55,187,242	55,187,903	11	0.037	+	55,187,242	55,187,903	11	0.017	+
X	*CLCN5*	Chloride voltage-gated channel 5	49,687,028	49,687,610	11	0.037	+	49,687,028	49,687,822	13	0.011	+
X	*PGK1*	Phosphoglycerate kinase 1	77,359,355	77,359,749	11	0.039	+	77,359,355	77,359,924	13	0.013	+
X	*ACSL4*	Acyl-CoA synthetase long chain family member 4	108,976,035	108,976,893	10	0.039	+	108,976,035	108,976,893	10	0.019	+
X	*NUP62CL* *CXorf41 (PIH1D3)*	Nucleoporin 62 C-terminal like PIH1 domain containing 3	106,449,457	106,449,904	11	0.040	+	106,449,457	106,449,944	12	0.018	+
X	*YIPF6*	Yip1 domain family member 6	67,718,299	67,719,066	11	0.041	+	67,718,299	67,719,066	11	0.018	+
X	*MSL3*	MSL complex subunit 3	11,776,256	11,776,935	8	0.041	+	11,776,256	11,776,935	8	0.024	+
X	*ZNF182* *SPACA5*	Zinc finger protein 182 Sperm acrosome associated 5	47,862,977	47,863,707	11	0.042	+	47,862,977	47,863,707	11	0.027	+
X	*KIF4A*	Kinesin family member 4A	69,509,686	69,510,172	11	0.042	+	69,509,542	69,510,172	12	0.016	+
X	*ATP6AP1*	ATPase H + transporting accessory protein 1	153,656,771	153,657,411	9	0.044	+	153,656,771	153,657,411	9	0.021	+
X	*SYP*	Synaptophysin	49,056,505	49,056,886	10	0.044	+	49,056,505	49,057,013	11	0.019	+
X	*TSPYL2*	Testis-specific protein Y-encoded like 2	53,111,116	53,112,003	10	0.044	+	53,111,116	53,112,003	10	0.025	+
X	*EDA*	Ectodysplasin A	68,835,678	68,836,202	10	0.044	+	68,835,729	68,836,202	9	0.028	+
X	*PPP1R3F*	Protein phosphatase 1 regulatory subunit 3F	49,126,087	49,126,486	9	0.046	+	49,126,087	49,126,486	9	0.024	+
X	*LONRF3*	LON peptidase N-terminal domain and ring finger 3	118,108,506	118,109,413	10	0.047	+	118,108,506	118,109,413	10	0.028	+
Girls												
X	*HTATSF1*	HIV-1 tat specific factor 1	135,578,793	135,579,487	13	0.018	+	135,578,793	135,580,181	19	0.002	+
6	NA	NA	31,650,735	31,651,094	14	0.010	+	31,650,735	31,651,029	11	0.029	+
1	NA	NA	152,161,237	152,162,025	7	0.016	−	152,161,237	152,162,025	7	0.027	−
5	*RUFY1*	RUN and FYVE domain containing 1	178,986,131	178,986,906	9	0.035	+	178,986,291	178,986,906	8	0.049	+
X	*XIAP*	X-linked inhibitor of apoptosis	122,993,695	122,994,071	11	0.032	+	122,993,419	122,994,071	13	0.005	+

The overlapping DMRs refer to both complete (same start and end site) and partial (different start and/or end site) overlaps. Start, end are chromosomal coordinates according to genome built 37; Dir. refers to the direction of association with prenatal As or IGFBP3 concentrations; L, the number of CpG sites within the DMR. Cytogenic band enrichment analysis for the genes corresponding to the overlapping DMRs in boys indicated significant enrichment (FDR-adjusted *p* value < 0.05) for chromosome X in Xp11.23 (*CLCN5, SPACA5, SYP, PPP1R3F*), Xq28 (*DNASE1L1, HMGB3, ATP6AP1, IKBKG*) and Xq24 (*UPF3B, RNF113A, LONRF3*)

## Discussion

In the present study, we found that increasing maternal urinary arsenic concentrations in early pregnancy was associated with higher plasma concentrations of IGFBP3 in the children at 9 years of age. To the best of our knowledge, this is the first epidemiological evidence that inorganic As may influence plasma IGFBP3 concentrations. Importantly, the children’s concurrent urinary arsenic did not appear to influence IGFBP3. Our results indicate that the associations with IGFBP3 are, at least in part, mediated by changes in DNA methylation patterns at both CpG and region (DMR) level. In addition, the associations between maternal urinary As and IGFBP3 were linear even at low exposure levels.

In vitro and in vivo studies have reported alterations of *IGFBP3* gene expression by arsenic. Exposure to sodium arsenite decreased gene expression of *IGFBP3* in A549 human lung cancer cells (van Breda et al. 2015) and human normal (hEp), premalignant (SCC12F2) and malignant (SCC9) keratinocytes (Rea et al. 2003). Similar to our findings, in utero exposure to sodium arsenite in mice combined with repeated postnatal exposure (post-weaning) to tetradecanoylphorbol acetate (a tumor promoter) resulted in increased *IGFBP3* gene expression in the liver 21 weeks post-weaning (Liu et al. 2006). Overall, based on the literature and our study, inorganic As seems to interact with IGFBP3, but the degree and direction of effect may be time-dependent and tissue-specific and further studies are need to elucidate this interaction.

We did not find an association between exposure to As (prenatal or concurrent) and plasma concentration of IGF1, a marker of growth that mediates bone, skeletal muscle and cartilage development, at 9 years of age. Similarly, we did not observe any association between exposure to As and the molar ratio IGF1/IGFBP3, which stands for their inter-dependent biological function and relate to the tissue bioavailability of IGF1. This indicates that the associations observed between prenatal exposure to As and IGFBP3 are likely independent of IGF1, and consequently, not necessarily related to effects on growth and development. Indeed, molecular studies have shown that IGFBP3 has biological functions that are independent of IGF1, such as involvement in tumor development and progression by mediating DNA damage responses, autophagy and apoptosis (Baxter 2013; Jogie-Brahim et al. 2009). Epidemiological studies have yet to show if there are any associations between circulating IGFBP3 concentrations and cancer risk or prognosis (Baxter 2014). *IGFBP3* expression has been reported to be increased in head and neck squamous cell carcinomas as well as renal clear cell carcinoma. For breast cancer tissue, the results are conflicting and in some cases high mRNA levels seem to be associated with a favourable prognostic (reviewed in Baxter 2014). In addition, epidemiological studies have found a positive association between the risk of developing type 2 diabetes mellitus and circulating IGFBP3 concentrations (Drogan et al. 2016; Rajpathak et al. 2012), with some evidence that these effects are independent of IGF1 (Drogan et al. 2016). A positive effect of As on IGFBP3 concentrations could potentially explain in part the epidemiological association between exposure to As and type 2 diabetes that was put forward by a meta-analysis (Wang et al. 2014).

We found certain sex differences in the characteristics of our study sample boys had lower IGF1 and IGFBP3 as compared with girls both in the whole cohort and the EWAS subset; similar sex differences have previously been reported (Casazza et al. 2008). Boys also showed more DMPs and DMRs associated with As compared with girls. This is consistent with a previous study on the same cohort where prenatal As was associated with more changes in DNA methylation at a CpG level in the cord blood of boys and biological pathways related to cancer development were overrepresented in boys, but not in girls (Broberg et al. 2014). In addition, prenatal exposure to As was associated with global changes in cord blood DNA methylation with indications for sex-dependent differences in another Bangladeshi cohort (Pilsner et al. 2012). In our study, circulating IGFBP3 concentrations were negatively associated with methylation of the cg16447589 in boys and positively in girls. Similar sex differences were also observed for the association between prenatal As and methylation of this CpG. Interestingly, we also observed an enrichment in differentially methylated regions on the X chromosome in boys, but not in girls, which could potentially explain an increased susceptibility of As-induced changes in DNA methylation in this sex, as they only bear one copy of chromosome X.

There is emerging evidence linking epigenetic changes, such as alterations in DNA methylation, to the mechanisms behind certain As-induced toxicity [reviewed in (Argos 2015; Ren et al. 2011)]. DNA methylation has a high plasticity in utero, making embryogenesis a particularly susceptible window for epigenetic alterations that can alter disease susceptibility later in life (Hochberg et al. 2011). In general, the correlation between DNA methylation and gene expression of nearby genes in studies focused on As was reported to be low (Boellmann et al. 2010; Engstrom et al. 2017; Rojas et al. 2015). However, changes in DNA methylation could have a permissive role in regulating gene expression by, e.g. altering chromatin conformation (Boellmann et al. 2010). In the present study, we found regulatory regions containing binding motifs for transcription factors that were particularly enriched within the DMRs, and were mostly hypermethylated. Interestingly, our top DMR associated with both prenatal As and plasma IGFBP3 in a regulatory region and was located on *MIR886*. MIR886 was reported to be a tumor suppressor, for which methylation is inversely correlated with expression and is predictive of outcome in acute myeloid leukaemia (Treppendahl et al. 2012).

The strengths of our study include the population-based prospective study design, relatively large sample size for evaluating associations between As and IGF1 and IGFBP3, available information on important covariates that could act as confounders (e.g. nutritional and socioeconomic status, recent infection status), individual As exposure assessment based on urinary As concentrations during early pregnancy and at 9 years of age. An additional strength of our study is that we found associations between changes in DNA methylation and a phenotype (circulating IGFBP3 amounts) which can bring light into understanding the underlying mechanisms for As toxicity. Furthermore, the DNA methylation analyses were adjusted for estimated cell counts which is regarded as a potential source of confounding (Argos 2015). One limitation of the study is that we have evaluated IGFBP3 in plasma at only one time-point (9 years) and therefore we cannot assess whether the association with prenatal As exposure was present already at birth. Another limitation of this study is the fact that DNA methylation was only performed in a subset sample, selected based on DNA quality. The lower number of individuals reduced the power of the DNA methylation analysis and therefore of the mediation analysis.

In conclusion, maternal arsenic exposure in early pregnancy appeared to affect the circulating IGFBP3 in school-aged Bangladeshi children. We found evidence that this association was likely partly mediated by differential DNA methylation of both individual CpG sites and DNA regions. It is of importance to follow-up on these observations and clarify whether such changes in circulating IGFBP3 levels could influence susceptibility of developing type 2 diabetes and modulate cancer risk later in life. The indicated sex differences should be interpreted with caution due to the low number of individuals following stratification by sex (especially in the EWAS sub-sample).

## Electronic supplementary material

Below is the link to the electronic supplementary material.

Supplementary material 1 (PDF 961 KB)
