# Supplementary material for: Prenatal arsenic exposure is associated with increased plasma IGFBP3 concentrations in 9-year-old children partly via changes in DNA methylation
Source: Arch Toxicol. 2018 Jun 8;92(8):2487–500. doi: 10.1007/s00204-018-2239-3 (PMC6063321; doi:10.1007/s00204-018-2239-3)
Supplement: Supplementary file 1 — Supplementary material 1 (PDF 961 KB) [file 204_2018_2239_MOESM1_ESM.pdf]

SUPPLEMENTAL MATERIAL

Prenatal arsenic exposure is associated with increased plasma IGFBP3 concentrations in 9-year-old children partly via changes in DNA methylation

Anda R. Gliga[1], Karin Engström[2], Maria Kippler[1], Helena Skröder[1], Sultan Ahmed[3], Marie Vahter[1], Rubhana Raqib[3], Karin Broberg[1]

[1]Unit of Metals and Health, Institute of Environmental Medicine, Karolinska Institutet, Stockholm, Sweden

[2]Division of Occupational and Environmental Medicine, Lund University, Lund, Sweden

[3]Division of Infectious Diseases, icddr,b, Dhaka, Bangladesh

TABLE OF CONTENTS

page

Table S1……3

Table S2……5

Table S3……6

Table S4……8

Table S5……9

Table S6……10

Figure S1…… 13

Table S1. Linear regression analysis of methylation of CpG sites within the *IGFBP3* gene in mononuclear cells at 9 years with IGFBP3 concentrations in plasma.[a]

CpG	Location in *IGFBP3*	All children (n=111) β (95% CI); p-value	Boys (n=48) β (95% CI); p-value	Girls (n=63) β (95% CI); p-value
cg16460681	promoter	56.88 (-30.05, 143.81); 0.197	101.30 (-51.00, 253.55); 0.187	41.62 (-65.30, 148.53); 0.439
cg00419512	promoter	-45.36 (-148.39, 56.78); 0.385	-57.20 (-167.59, 166.45); 0.995	-71.40 (-208.64, 65.84); 0.302
cg00118103	promoter	75.77 (-41.99, 193.45), 0.205	65.36 (-124.92, 255.65); 0.492	82.02 (-70.21, 234.25); 0.285
cg08541297	promoter	39.35 (-29.36, 108.07); 0.259	-88.58 (-205.00, 27.84); 0.132	99.54 (16.11, 182.97); 0.0202
cg04690927	promoter	64.99 (-36.28, 166.27); 0.206	-52.57 (-202.76, 97.59); 0.484	152.00 (17.73, 286.32); 0.0272
cg22403266	promoter	6.57 (-64.58, 77.72); 0.855	-15.89 (-125.95, 94.18); 0.772	19.15 (-76.41, 114.71); 0.689
cg04727332	promoter	50.50 (-92.78, 193.78); 0.486	6.26 (-207.28, 219.81); 0.953	69.43 (-134.08, 272.94); 0.497
cg04796162	promoter	12.11 (-38.50, 62.73); 0.636	-26.09 (-100.62, 48.44); 0.484	40.08 (-31.59, 111.75); 0.267
cg13977557	promoter	51.58 (-125.68, 229.43); 0.564	305.20 (26.82, 583.66); 0.0324	-114.70 (-352.14, 122.79); 0.338
cg12244052	promoter	-15.22 (-63.31, 32.88); 0.532	-63.58 (-143.57, 16.40); 0.116	7.25 (-55.85, 70.36); 0.819
cg10094651	promoter	14.43 (-25.23, 54.10); 0.472	-0.57 (-58.47, 57.33); 0.984	25.86 (-31.78, 83.50); 0.373
cg05083496	promoter	8.84 (-26.97, 44.61); 0.626	21.87 (-30.68, 74.43); 0.406	-4.22 (-56.56, 48.11); 0.872
cg23455440	promoter	26.35 (-34.28, 86.98); 0.391	-12.56 (-125.97, 100.84); 0.824	44.27 (-31.08, 119.62); 0.245
cg15208757	promoter	21.81 (-50.37, 94.00); 0.550	-65.69 (-189.04, 57.66); 0.289	66.85 (-25.89, 159.58); 0.155
cg24772240	promoter	4.03 (-53.26, 61.35); 0.889	-6.51 (-109.12, 96.10); 0.898	7.37 (-63.34, 78.09); 0.835
cg07910986	promoter	-5.35 (-82.45, 71.74); 0.891	-24.61 (-139.93, 90.70); 0.669	-3.00 (-114.96, 108.96); 0.957
cg16447589	promoter	-4.35 (-41.31, 32.60); 0.816	-61.47 (-128.03, 5.08); 0.0694	21.65 (-24.27, 67.57); 0.349
cg09619271	promoter	16.58 (-55.91, 89.07); 0.651	-13.32 (-126.71, 100.08); 0.814	26.25 (-73.46, 125.95); 0.600
cg19536059	promoter	-9.87 (-89.18, 69.45); 0.806	-59.00 (-179.20, 61.20); 0.328	16.95 (-93.84, 127.74); 0.761
cg08831744	promoter	16.87 (-52.46, 86.22); 0.630	-14.73 (-111.81, 82.35); 0.761	62.84 (-48.68, 174.37); 0.264
cg20351904	promoter	-10.88 (-122.55, 100.78); 0.847	-130.10 (-307.77, 47.55); 0.147	59.52 (-96.40, 215.44); 0.448
cg06789764	promoter	7.87 (-66.11, 81.86); 0.833	118.20 (6.01, 230.34); 0.039	-63.67 (-160.20, 32.85); 0.1919
cg24901098	promoter	-1.90 (-102.31, 98.50); 0.970	-71.53 (-218.93, 75.87); 0.333	70.76 (-72.58, 214.10); 0.327
cg10677697	promoter	2.60 (-89.00, 94.19); 0.955	43.89 (-94.93, 182.70); 0.527	-40.02 (-164.44, 84.39); 0.522
cg22800581	promoter	32.23 (-119.92, 184.37); 0.675	-73.40 (-300.11, 153.30); 0.517	128.50 (-86.62, 343.70); 0.237
cg06713098	promoter	4.81 (-42.71, 52.35); 0.841	-21.41 (-93.66, 50.85);0.554	24.19 (-40.37, 88.76); 0.456
cg05867388	promoter	-27.05 (-140.22, 86.12); 0.637	26.15 (-131.49, 183.81); 0.740	-83.75 (-248.58, 81.09); 0.313
cg26434048	promoter	3.52 (-34.91, 41.95); 0.856	-75.73 (-142.78, -8.67); 0.0278	35.12 (-11.05, 81.29); 0.133
cg12902183	intragenic	-0.17 (-23.48, 23.14); 0.988	6.73 (-36.34, 49.82); 0.7541	-0.40 (-30.19, 29.38); 0.979

cg16875425	intragenic	35.99 (0.85, 71.13); 0.0448	4.30 (-51.28, 59.88); 0.877	66.72 (20.10, 113.34); 0.0058
cg03776080	intragenic	91.76 (32.54, 150.96); 0.0027	-14.25 (-139.63, 111.13); 0.820	126.00 (60.86, 191.18); 0.0003
cg23193639	intragenic	57.77 (-2.71, 118.25); 0.061	16.21 (-79.78, 112.22); 0.735	85.36 (4.76, 165.94); 0.0383
cg22083798	intragenic	36.78 (1.47, 72.09); 0.0413	19.46 (-35.82, 74.75); 0.482	47.44 (-1.92, 96.80); 0.0592
cg24942272	intragenic	96.77 (-23.00, 216.54); 0.112	13.08 (-177.09, 203.26); 0.890	13.87 (-24.26, 301.63); 0.938
cg02120774	intragenic	80.50 (-7.14, 168.15); 0.0714	-126.10 (-265.85, 13.72); 0.076	196.60 (91.29, 301.83); 0.0004
cg11753867	intragenic	100.50 (-37.31, 238.25); 0.151	225.30 (6.80, 443.78); 0.0435	34.45 (-148.37, 217.28); 0.707
cg20850023	intragenic	56.35 (-8.58, 121.29); 0.0882	-38.52 (-144.96, 67.91); 0.470	12.31 (38.82, 207.31); 0.0049
cg21268650	intragenic	43.59 (-14.26, 101.44); 0.138	30.67 (-60.77, 122.11); 0.503	54.43 (-24.27, 133.13); 0.172
cg24381682	intragenic	-65.91 (-147.94, 16.12); 0.114	-73.32 (-212.58, 65.93); 0.294	-66.10 (-173.32, 41.12); 0.222

[a] Methylation data was derived from the ComBat adjusted % methylation values (excluding sites from X and Y chromosomes for analysis on all children and including sites from X and Y chromosomes for the sex-stratified analysis). The linear models were adjusted for child sex (except when stratified for sex), estimated granulocytes and monocytes.

Table S2. Number of differentially methylated positions (DMPs) and differentially methylated regions (DMRs) in peripheral blood mononuclear cells at 9 years correlated with maternal urinary arsenic (GW 8, ln-transformed) or to IGFBP3 concentrations in plasma at 9 years.[a]

	**Prenatal arsenic**	**IGFBP3**	**Overlap Top 2000**
**#DMPs**			
All (n=112/111)	9	0	14
Boys (n=49/48)	57	1162	11
Girls (n=63/63)	15	11	11
**#DMRs**			
All (n=112/111)	42	45	10
Boys (n=49/48)	39	109	26
Girls (n=63/63)	12	262	5

[a]DMPs for which q values < 0.05 (FDR adjusted p-values) are considered as differentially methylated. The differential methylation was performed using linear models for microarray data (*limma* package), adjusted for sex (unless stratified for sex), estimated granulocytes and monocytes. The overlap between the two analyses was performed considering the top 2000 differentially methylated sites. DMRs were generated using the *bumphunter* function within the *minfi* package. The overlap between the two analyses was performed considering both complete and partial region overlap. n, number of children for which the differential methylation analysis was performed

Table S3. Differentially mediated CpG sites in peripheral blood mononuclear cells at 9 years in relation to maternal urinary As (GW 8, ln-transformed)[a]

				Prenatal arsenic		
	ID	Chr	Gene symbol	β	p	q
ALL (n=112)						
	cg01718785	7	*PTPRN2*	0.239	< 0.001	0.013
	cg14855326	10	*BTRC*	0.470	< 0.001	0.024
	cg14525832	14	*ALKBH1 (C14orf156)*	-0.333	< 0.001	0.037
	cg09652472	7	*KIAA1908*	0.104	< 0.001	0.037
	cg20565833	4	*FAM190A*	0.198	< 0.001	0.049
	cg15491540	20	*SPTLC3*	0.297	< 0.001	0.049
	cg04848823	16	*NA*	0.206	< 0.001	0.049
	cg08452525	10	*C10orf18*	-0.254	< 0.001	0.049
	cg22922226	14	*C14orf132*	0.099	< 0.001	0.049
BOYS (n=49)						
	cg09857273	8	*CDH17*	0.215	< 0.001	0.004
	cg04933829	14	*NA*	0.259	< 0.001	0.004
	cg16052901	6	*VPS52 (RPS18)*	0.199	< 0.001	0.004
	cg12535476	6	*BMP5*	0.224	< 0.001	0.006
	cg15778745	13	*SLITRK5*	-0.463	< 0.001	0.006
	cg23202738	3	*C3orf15*	-0.220	< 0.001	0.006
	cg13002167	7	*NA*	0.212	< 0.001	0.006
	cg21003654	4	*JAKMIP1*	-0.136	< 0.001	0.006
	cg23500924	5	*NA*	0.286	< 0.001	0.006
	cg00298324	20	*FAM110A*	-0.194	< 0.001	0.006
	cg23085143	7	*SVOPL*	-0.350	< 0.001	0.006
	cg01609139	4	*NA*	-0.460	< 0.001	0.006
	cg05658107	4	*RHOH*	-0.393	< 0.001	0.013
	cg21222426	9	*NA*	-0.288	< 0.001	0.016
	cg07520506	12	*CD163L1*	-0.343	< 0.001	0.018
	cg14855326	10	*BTRC*	0.489	< 0.001	0.018
	cg16447589	7	*IGFBP3*	-0.239	< 0.001	0.020
	cg06241901	6	*TTBK1*	-0.127	< 0.001	0.021
	cg20610452	2	*NA*	-0.175	< 0.001	0.021
	cg01567825	7	*DOCK4*	-0.228	< 0.001	0.021
	cg05371749	16	*ITPRIPL2*	-0.175	< 0.001	0.021
	cg02818189	1	*NA*	-0.204	< 0.001	0.022
	cg03735888	19	*ZNF132*	-0.321	< 0.001	0.024
	cg18899777	8	*GRHL2*	-0.218	< 0.001	0.024
	cg18806997	1	*SNORD46 (RPS8)*	0.172	< 0.001	0.024
	cg01015123	3	*OTOL1*	0.209	< 0.001	0.025
	cg14439249	6	*NA*	0.193	< 0.001	0.025
	cg16332700	10	*GPR123*	0.134	< 0.001	0.025
	cg13308137	11	*CUGBP1*	-0.165	< 0.001	0.025
	cg24789682	4	*NA*	0.211	< 0.001	0.025
	cg13906874	11	*NA*	0.426	< 0.001	0.025
	cg17519280	5	*ATG12*	0.240	< 0.001	0.028
	cg10599444	14	*MMP14*	-0.114	< 0.001	0.030
	cg04634427	15	*NA*	-0.284	< 0.001	0.030
	cg06984073	5	*SV2C*	-0.197	< 0.001	0.030
	cg21548021	8	*NA*	0.237	< 0.001	0.033
	cg12127706	11	*CTTN*	-0.192	< 0.001	0.033
	cg11793332	13	*ELF1*	-0.195	< 0.001	0.033
	cg10215401	8	*NA*	-0.236	< 0.001	0.033
	cg11299264	19	*NA*	-0.124	< 0.001	0.033
	cg20963741	13	*NA*	-0.119	< 0.001	0.033
	cg24246628	6	*KIF25*	-0.502	< 0.001	0.033

	cg11414507	12	*NA*	-0.123	< 0.001	0.033
	cg17852326	1	*HLX*	0.112	< 0.001	0.038
	cg03839794	14	*NPAS3*	-0.513	< 0.001	0.042
	cg22707529	6	*PHACTR2*	-0.178	< 0.001	0.043
	cg23326536	13	*EDNRB*	0.203	< 0.001	0.043
	cg08918658	6	*SYNJ2*	-0.144	< 0.001	0.043
	cg20187049	3	*NA*	0.195	< 0.001	0.043
	cg01834305	5	*AACSL*	0.172	< 0.001	0.045
	cg00478710	3	*NA*	-0.111	< 0.001	0.045
	cg03960811	2	*ZNF385B*	0.298	< 0.001	0.048
	cg25621286	5	*NA*	0.134	< 0.001	0.049
	cg01913577	1	*NA*	0.194	< 0.001	0.050
	cg20705392	17	*NTN1*	-0.152	< 0.001	0.050
	cg00320288	16	*NA*	-0.112	< 0.001	0.050
	cg15310415	6	*NA*	0.477	< 0.001	0.050
**GIRLS (n=63)**						
	cg15576338	1	*NA*	0.213	< 0.001	0.005
	cg14525832	14	*ALKBH1 (C14orf156)*	-0.468	< 0.001	0.013
	cg20009461	17	*FOXK2*	0.279	< 0.001	0.013
	cg03169856	8	*FER1L6*	0.265	< 0.001	0.013
	cg22139563	17	*KRT38*	0.300	< 0.001	0.013
	cg15487992	19	*UBL5*	-0.268	< 0.001	0.013
	cg00804031	19	*PIAS4*	-0.416	< 0.001	0.013
	cg13753209	17	*CLTC*	-0.244	< 0.001	0.013
	cg03426602	2	*NA*	0.243	< 0.001	0.016
	cg21725754	4	*NAF1*	-0.376	< 0.001	0.017
	cg04575016	3	*C3orf64*	-0.213	< 0.001	0.023
	cg20608709	15	*ADAM10*	0.516	< 0.001	0.027
	cg21101726	1	*REN*	0.262	< 0.001	0.033
	cg25311330	1	*TMEM183A (TMEM183B)*	-0.498	< 0.001	0.043
	cg16105461	11	*DAGLA*	0.118	< 0.001	0.048

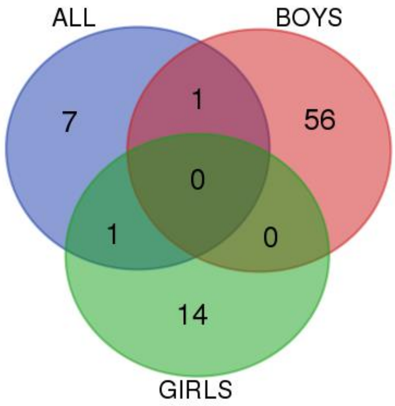

Lower insert is a Venn diagram indicating the number of overlapping CpGs between all individuals, boys and girls

[a]β – linear regression estimates from the differential methylation analysis adjusted for sex (unless stratified for sex), estimated granulocytes and monocytes; input data were ComBat adjusted M-values; q – FDR-adjusted p-value

Table S4. Differentially methylated positions in peripheral blood mononuclear cells at 9 years in relation to maternal urinary As (GW 8, ln-transformed) and plasma IGFBP3 concentrations[a]

Top 2000 overlap					Prenatal As			IGFBP3		
	ID	Chr	Gene	Location	Dir.	p	q	Dir.	p	q
**ALL**										
	cg00150520	1	RNF220	intragenic	+	< 0.005	0.435	+	< 0.005	0.454
	cg02250992	12	SCARB1	intragenic	+	< 0.005	0.450	+	< 0.005	0.371
	cg02824980	19	CYP4F12	promoter	+	< 0.005	0.493	+	< 0.005	0.401
	cg04912993	3	ALCAM	intragenic	+	< 0.005	0.450	+	< 0.005	0.287
	cg04961397	16	SETD1A	intragenic	+	< 0.005	0.426	+	< 0.005	0.421
	cg08292120	21	NA	intergenic	+	< 0.005	0.401	+	< 0.005	0.312
	cg08570472	2	GAREML	intragenic	+	< 0.005	0.511	+	< 0.005	0.362
	cg10716835	10	CYP26A1	intragenic	+	< 0.005	0.433	+	< 0.005	0.329
	cg10856894	6	PDE10A	intragenic	+	< 0.005	0.513	-	< 0.005	0.362
	cg13316171	2	ZC3H15	promoter	+	< 0.005	0.519	+	< 0.005	0.402
	cg23713249	7	GIMAP1	intragenic	+	< 0.005	0.528	+	< 0.005	0.395
	cg24312661	15	SEMA4B	promoter	+	< 0.005	0.472	+	< 0.005	0.464
	cg26144999	13	NA	intergenic	+	< 0.005	0.411	-	< 0.005	0.371
	cg26163223	1	MUL1	promoter	+	< 0.005	0.467	+	< 0.005	0.419
**BOYS**										
	cg01441988	22	KIAA0930	intragenic	-	< 0.005	0.262	-	< 0.005	0.036
	cg01929855	4	JAKMIP1	intragenic	-	< 0.005	0.243	-	< 0.005	0.050
	cg06507551	7	CTTNBP2	promoter	-	< 0.005	0.215	-	< 0.005	0.058
	cg12844324	8	CHD7	intragenic	-	< 0.005	0.197	-	< 0.005	0.058
	cg12849291	2	SEMA4C	promoter	+	< 0.005	0.205	+	< 0.005	0.066
	cg14075624	6	C6orf136	intragenic	+	< 0.005	0.272	+	< 0.005	0.032
	cg14622879	6	NA	intergenic	+	< 0.005	0.246	+	< 0.005	0.041
	cg16420756	6	NA	intergenic	-	< 0.005	0.267	-	< 0.005	0.045
	cg22193726	12	PTPN11	intragenic	+	< 0.005	0.208	+	< 0.005	0.028
	cg22778120	22	PRR5	intragenic	-	< 0.005	0.248	-	< 0.005	0.034
	cg24712249	1	SYT11	intragenic, promoter	+	< 0.005	0.192	+	< 0.005	0.049
**GIRLS**										
	cg01528321	10	TSPAN14	intragenic	-	< 0.005	0.339	+	< 0.005	0.349
	cg02057681	3	CHL1	intragenic	+	< 0.005	0.455	-	< 0.005	0.327
	cg14185808	2	MRPS9	promoter	-	< 0.005	0.484	+	< 0.005	0.316
	cg15450139	12	ARHGDIB	promoter	-	< 0.005	0.352	+	< 0.005	0.214
	cg16758084	3	CACNA2D3	3'UTR	+	< 0.005	0.191	-	< 0.005	0.353
	cg18326022	17	ITGB4	intragenic, promoter	+	< 0.005	0.431	+	< 0.005	0.363
	cg22016716	6	SUPT3H	intragenic	+	< 0.005	0.453	-	< 0.005	0.344
	cg24336278	1	TP73	promoter	-	< 0.005	0.169	+	< 0.005	0.327
	cg26144999	13	NA	intergenic	+	< 0.005	0.399	-	< 0.005	0.269
	cg26612156	13	NA	intergenic	-	< 0.005	0.463	+	< 0.005	0.247
	cg26810336	X	IL1RAPL1	promoter	+	< 0.005	0.385	+	< 0.005	0.339

[a]Table contains the overlaps from the top 2000 DMPs for the two analyses. The differential methylation analysis was adjusted for sex (unless stratified for sex), estimated granulocytes and monocytes; input data were ComBat-adjusted M-values; Dir. refers to the direction of the association; q – FDR-adjusted p-value

Table S5. Mediation regression analyses[a]

Top 2000 overlap					Prenatal As versus IGFBP3 (9 years)		% Mediation
	ID	Chr	Gene	Location	β	p-value	
**ALL (n=524) study sample**							
MVA model					76	0.009	
**ALL (n=110) EWAS sub-set**							
MVA model					85	0.257	
MVA model +	cg00150520	1	*RNF220*	intragenic	28	0.702	67%
+	cg02250992	12	*SCARB1*	intragenic	46	0.539	46%
+	cg02824980	19	*CYP4F12*	promoter	37	0.622	56%
+	cg04912993	3	*ALCAM*	intragenic	43	0.577	49%
+	cg04961397	16	*SETD1A*	intragenic	23	0.769	73%
+	cg08292120	21	*NA*	intergenic	34	0.643	60%
+	cg08570472	2	*GAREML*	intragenic	45	0.548	47%
+	cg10716835	10	*CYP26A1*	intragenic	24	0.744	72%
+	cg10856894	6	*PDE10A*	intragenic	152	0.047	-79%
+	cg13316171	2	*ZC3H15*	promoter	31	0.668	64%
+	cg23713249	7	*GIMAP1*	intragenic	34	0.661	60%
+	cg24312661	15	*SEMA4B*	promoter	36	0.638	58%
+	cg26144999	13	*NA*	intergenic	125	0.097	-47%
+	cg26163223	1	*MUL1*	promoter	14	0.849	84%
**BOYS (n=261) study sample**							
MVA model					85	0.024	
**BOYS (n=47) EWAS sub-set**							
MVA model					152	0.188	
MVA model +	cg01441988	22	*KIAA0930*	intragenic	32	0.762	79%
+	cg01929855	4	*JAKMIP1*	intragenic	51	0.654	66%
+	cg06507551	7	*CTTNBP2*	promoter	94	0.397	38%
+	cg12844324	8	*CHD7*	intragenic	61	0.604	60%
+	cg12849291	2	*SEMA4C*	promoter	78	0.464	49%
+	cg14075624	6	*C6orf136*	intragenic	79	0.44	48%
+	cg14622879	6	*NA*	intergenic	72	0.507	53%
+	cg16420756	6	*NA*	intergenic	46	0.668	70%
+	cg22193726	12	*PTPN11*	intragenic	23	0.842	85%
+	cg22778120	22	*PRR5*	intragenic	80	0.456	47%
+	cg24712249	1	*SYT11*	promoter	16	0.89	89%
+	cg16447589	7	*IGFBP3*	promoter	121	0.311	20%
**GIRLS (n=263) study sample**							
MVA model					76	0.082	
**GIRLS (n=63) EWAS sub-set**							
MVA model					11	0.918	

[a]Linear regression analysis maternal urinary As (GW 8, ln-transformed) with plasma IGFBP3 (9 years) on the sub-set of individuals for which methylation analysis was performed. In the multi-variable adjusted model (MVA) we considered child sex (except when stratified for sex), plasma CRP (9 years) and plasma IGF1 as covariates. In addition, we evaluated the effect of potential epigenetic mediators by adding them one by one (ComBat-adjusted % methylation) as covariates in the MVA regression model. The mediator variables were identified from the top 2000 CpG sites that were associated both with the exposure (ln transformed As in urine, GW8) and the outcome (IGFBP3 ng/mL 9 years) in the respective differential methylation EWAS analyses. For boys we tested an additional potential mediator (cg16447589) identified on the promoter region of the *IGFBP3*. % mediation was calculated as $(\beta_{(MVA)} - \beta_{(MVA+\%methylationCpG)})/\beta_{(MVA)}$. The mediation analysis could not be performed for girls due to the low estimates in the EWAS sub-set compared to the whole sample.

Table S6. Differentially mediated regions in peripheral blood mononuclear cells at 9 years in relation to maternal urinary As (GW 8, ln-transformed)[a]

Prenatal As								
	**Chr**	**Gene symbol**	**Start**	**End**	**L**	**Dir.**	**p-value**	**q-value**
**ALL**								
	5	MIR886	135415693	135416613	16	+	0.001	0.318
	22	GSTT1	24384105	24384573	11	-	0.001	0.318
	17	SLFN12	33759484	33760419	12	+	0.002	0.364
	6	NA	29648271	29649084	20	-	0.003	0.364
	17	ALOX12	6899085	6899758	12	-	0.005	0.364
	5	FLJ44606	126408756	126409372	11	-	0.005	0.364
	5	RUFY1	178986131	178986906	9	+	0.007	0.364
	6	PSMB9	32822911	32823292	4	+	0.008	0.364
	11	CALCA	14993378	14994045	13	-	0.009	0.364
	6	NA	28601269	28601477	12	+	0.009	0.364
	16	ABAT	8806531	8807018	10	-	0.010	0.364
	11	C11orf21	2322500	2322935	13	-	0.010	0.364
	1	NA	152161237	152162025	7	-	0.011	0.364
	5	PCDHB7	140552347	140552600	4	+	0.014	0.366
	1	OR2L13	248100345	248100614	7	-	0.015	0.366
	1	NA	147781827	147782558	7	+	0.017	0.374
	3	PLOD2	145879568	145879710	5	-	0.020	0.395
	1	PM20D1	205818956	205819492	7	+	0.021	0.395
	10	FGFR2	123355268	123356236	7	-	0.022	0.395
	22	C22orf27	31318103	31318546	9	-	0.024	0.395
	5	NA	1867978	1868639	5	+	0.025	0.395
	17	LOC728392	5402972	5403516	5	-	0.026	0.395
	7	NA	21208923	21209781	7	-	0.027	0.395
	19	AURKC	57742217	57742444	8	+	0.028	0.395
	12	NA	89748726	89748932	4	+	0.028	0.395
	7	PON1	94953770	94954202	7	+	0.029	0.395
	16	CLDN9	3062349	3062975	8	-	0.030	0.395
	11	LDHC	18433564	18433887	5	-	0.031	0.395
	6	TNXB	32064491	32064656	9	-	0.033	0.406
	4	NPFFR2	72897565	72897868	5	-	0.034	0.406
	1	S100A13	153599671	153600156	6	+	0.037	0.406
	4	JAKMIP1	6107021	6107649	7	-	0.038	0.406
	11	WT1	32449163	32450104	7	-	0.039	0.406
	5	CDX1	149546331	149546946	8	-	0.039	0.406
	5	SLC25A2	140683467	140683772	8	-	0.040	0.406
	2	DPP10	115419537	115420053	5	-	0.040	0.406
	11	NA	67383377	67383862	7	+	0.040	0.406
	6	NA	164506861	164507305	6	-	0.041	0.406
	4	GABRG1	46126066	46126448	7	-	0.041	0.406
	4	NA	4763259	4763372	4	-	0.045	0.411
	6	VPS52/RPS18	33240820	33241770	8	+	0.047	0.411
	11	CHORDC1	89956704	89957033	5	-	0.049	0.411
**BOYS**								
	5	MIR886	135415693	135416613	16	+	0.000	0.222

	6	TNXB	32063901	32064785	29	-	0.001	0.255
	X	BCYRN1	70712215	70712810	8	+	0.002	0.346
	22	GSTT1	24384105	24384573	11	-	0.003	0.456
	6	NA11	31650735	31651291	20	-	0.003	0.456
	X	NDUFB11/RBM10	47004051	47005131	18	+	0.006	0.534
	6	NA1	29648379	29649084	15	-	0.008	0.534
	X	IKBKG	153775262	153776009	16	+	0.010	0.534
	17	SLFN12	33759484	33760419	12	+	0.013	0.534
	X	RNF113A	119005413	119006122	14	+	0.014	0.534
	X	ZNF674	46404528	46405125	14	+	0.015	0.534
	5	PCDHB16	140562562	140563400	7	+	0.015	0.534
	5	FLJ44606	126408756	126409372	11	-	0.016	0.534
	X	UPF3B	118986897	118987328	13	+	0.019	0.534
	X	LOC100272228	149106454	149107029	13	+	0.021	0.534
	5	PCDHB11	140578896	140579644	9	+	0.024	0.534
	17	LOC728392	5402972	5403516	5	-	0.025	0.534
	2	LCLAT1	30669597	30669952	5	+	0.025	0.534
	X	TAZ/DNASE1L1	153639778	153640967	12	+	0.026	0.534
	X	HMGB3	150151580	150151823	11	+	0.029	0.534
	2	DPP10	115419537	115420053	5	-	0.035	0.534
	X	RPL36A	100645695	100646162	11	+	0.037	0.534
	X	FAM104B	55187242	55187903	11	+	0.037	0.534
	X	CLCN5	49687028	49687610	11	+	0.037	0.534
	X	PGK1	77359355	77359749	11	+	0.038	0.534
	5	PCDHGA2	140718731	140719302	5	+	0.039	0.534
	5	PCDHB7	140552347	140552600	4	+	0.039	0.534
	X	ACSL4	108976035	108976893	10	+	0.039	0.534
	X	NUP62CL/CXorf41	106449457	106449904	11	+	0.040	0.534
	X	YIPF6	67718299	67719066	11	+	0.041	0.534
	X	MSL3	11776256	11776935	8	+	0.041	0.534
	X	ZNF182/SPACA5	47862977	47863707	11	+	0.042	0.534
	X	KIF4A	69509686	69510172	11	+	0.042	0.534
	X	ATP6AP1	153656771	153657411	9	+	0.044	0.534
	X	SYP	49056505	49056886	10	+	0.044	0.534
	X	TSPYL2	53111116	53112003	10	+	0.044	0.534
	X	EDA	68835678	68836202	10	+	0.044	0.534
	X	PPP1R3F	49126087	49126486	9	+	0.046	0.534
	X	LONRF3	118108506	118109413	10	+	0.047	0.534
**GIRLS**								
	22	GSTT1	24384105	24384573	11	-	0.009	0.617
	6	NA	31650735	31651094	14	+	0.010	0.617
	1	OR2L13	248100276	248100614	8	-	0.011	0.617
	17	ALOX12	6899207	6899758	10	-	0.013	0.617
	1	NA	152161237	152162025	7	-	0.015	0.617
	X	HTATSF1	135578793	135579487	13	+	0.018	0.617
	19	HKR1	37825388	37825679	4	+	0.028	0.617
	12	SLC38A4	47219626	47219958	11	-	0.028	0.617
	X	XIAP	122993695	122994071	11	+	0.032	0.617
	5	RUFY1	178986131	178986906	9	+	0.035	0.617
	6	PSMB9	32822911	32823292	4	+	0.036	0.617

	5	FER	108083455	108083695	4	+	0.045	0.617

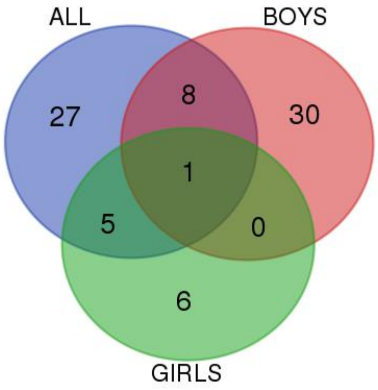

lower insert is a Venn diagram indicating the number of overlapping DMRs between all individuals, boys and girls

[a]*Start* and *End* are chromosomal coordinates according to genome built 37; L, number of CpG sites within the DMR; q-value, FDR-adjusted p-value; Dir, direction of association with Prenatal As

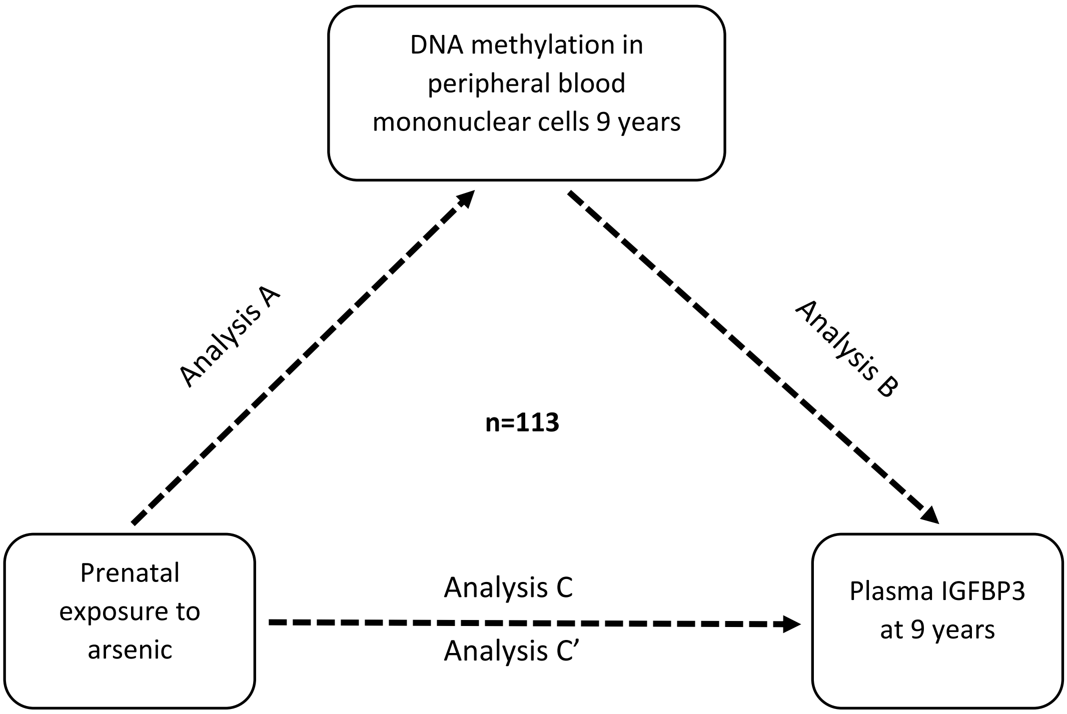

Figure S1. Mediation model

Analysis A evaluates the effect estimate of the association between prenatal exposure to arsenic and DNA methylation of CpG sites expressed as Combat-adjusted % methylation and Combat-adjusted M-values. Linear models were adjusted for child sex (except when stratified for sex), estimated granulocytes and monocytes. See Table 3 and Table S4.

Analysis B evaluates the effect estimate of the association between DNA methylation of CpG sites (expressed as Combat-adjusted % methylation and Combat-adjusted M-values) and plasma IGFBP3 concentrations. Linear models were adjusted for child sex (except when stratified for sex), estimated granulocytes and monocytes. See Table S1 and Table S4.

Analysis C evaluates the effect estimate of the association between prenatal exposure to arsenic and plasma concentrations of IGFBP3 at 9 years. Linear models were adjusted for child sex (except when stratified for sex), plasma CRP (9 years) and plasma IGF1. See Table S5.

Analysis C' evaluates the effect estimate of the association between prenatal exposure to arsenic and plasma concentrations of IGFBP3 at 9 years when the mediator is included as covariate. Linear models were adjusted similarly as for analysis C and, in addition, for DNA methylation of CpG sites of interest (one by one, expressed as Combat-adjusted % methylation values). See Table S5.
